# Edible insect larvae as sustainable sources of chitosan: Influence of species and drying method on functional and structural properties

**DOI:** 10.1016/j.fochx.2025.103133

**Published:** 2025-10-03

**Authors:** Ji Yoon Cha, Yea-Ji Kim, Jeong Heon Kim, Dong-Hyun Keum, Jaejoon Han, Yun-Sang Choi

**Affiliations:** a Research Group of Food Processing, Korea Food Research Institute, Wanju 55365, Republic of Korea; b Department of Biotechnology, Korea University, Seoul 02841, Republic of Korea

**Keywords:** edible insect, chitosan, correlation, physicochemical property, comparative analysis

## Abstract

Chitosan—a biopolymer produced by the deacetylation of chitin—finds numerous applications and is primarily obtained from the byproducts of marine crustacean processing. Given that this production method has numerous limitations, edible insects have drawn considerable attention as an alternative chitosan source. Herein, chitosan was extracted from the larvae of *Protaetia brevitarsis, Allomyrina dichotoma*, and *Tenebrio molitor* subjected to hot-air drying and freeze-drying, and its physicochemical and functional properties were compared with those of commercial shrimp-derived chitosan. Insect species and drying pretreatment influenced chitosan properties. Chitosan from hot-air-dried *A. dichotoma* showed the highest antioxidant activity, while that from *T. molitor* exhibited physicochemical and structural features comparable with those of shrimp-derived chitosan. In contrast, chitosan from hot-air-dried *P. brevitarsis* demonstrated a reduced solubility and deacetylation degree. These findings indicate that species- and process-dependent variations strongly affect functional performance and *A. dichotoma* and *T. molitor* are promising alternative chitosan sources.

## Highlights

• Chitosan is extracted from edible insect larvae dried using different methods.
• *Protaetia brevitarsis*, *Allomyrina dichotoma*, and *Tenebrio molitor* are used as insects.
• Properties of insect-derived chitosan are compared with those of commercial chitosan.
• Insect species and drying method significantly influence chitosan properties.
• Potential of insect-derived chitosan as a functional biomaterial is revealed.

## 1 Introduction

Chitosan is a natural biopolymer composed of D-glucosamine and *N*-acetyl-D-glucosamine and produced by the chemical or enzymatic deacetylation of chitin (Escobar Rodríguez et al., 2025; Shin et al., 2019), which is the second most abundant polysaccharide after cellulose and is found in crustaceans, insects, and fungi (Kumari et al., 2017). Owing to its biocompatibility, antimicrobial activity, and ecofriendly polyelectrolyte properties, chitosan finds numerous applications, including those in the food, pharmaceutical, biomedical, and environmental industries (Jiang et al., 2023; Wang et al., 2018; Zhang et al., 2022). Recent studies have emphasized the antioxidant, antimicrobial, and film-forming capacities of chitosan, highlighting its potential in active food packaging (Khatami et al., 2024; Malm et al., 2021). However, the conversion of chitin to chitosan can lead to depolymerization and, hence, the loss of certain polymer characteristics (Delezuk et al., 2011; da Silva et al., 2021). Processing conditions affect properties such as the deacetylation degree (DD), molecular weight, crystallinity, and viscosity of chitosan, thus influencing its performance (Triunfo et al., 2022). Despite the advances in chemical, enzymatic, and green chitosan extraction, large-scale production remains hindered by low scalability and cost efficiency (Röthig et al., 2023).

Owing to these challenges, edible insects have emerged as a sustainable alternative chitosan source (Kim et al., 2022). Compared with conventional livestock cultivation, insect cultivation requires fewer resources and produces less greenhouse gas emissions, thus representing an environmentally sustainable approach, while enabling the valorization of agricultural byproducts and thereby contributing to waste reduction and circular bioeconomy strategies (Fowles & Nansen, 2020; Franco et al., 2021; Lin et al., 2023). The insect cuticle contains 20 %–50 % chitin on a dry weight basis (da Silva et al., 2021) and may therefore be used to overcome the dependence on crustacean byproducts and the seasonal and geographical constraints of conventional chitosan sources (Escobar Rodríguez et al., 2025; Röthig et al., 2023). Recent studies have indicated that insect-derived chitosan may possess advantageous structural and biological properties and even exhibit antioxidant and antimicrobial activities comparable with or exceeding those of shrimp-derived chitosan (Ma et al., 2022). Despite the availability of studies on insect-derived chitosan, more integrated evaluations are needed to clarify variations across species and processing conditions and support the broader application potential of this material.

To explore the potential of insects as alternative chitosan sources, we herein extracted chitosan from the hot-air-dried and freeze-dried larvae of *Tenebrio molitor* (TM), *Protaetia brevitarsis* (PB), and *Allomyrina dichotoma* (AD) (Choi et al., 2024). Although TM has been extensively investigated, studies on PB and AD are limited, which highlights the need to expand research to these less explored species. The structural and physicochemical properties (including yield, DD, molecular weight, crystallinity, morphology, thermal stability, rheological behavior, and antioxidant activity) of different chitosan samples were systematically evaluated and compared with those of commercial shrimp-derived chitosan. Whereas most recent studies have emphasized application-oriented uses, the effect of pretreatment conditions on the structural and functional characteristics of insect-derived chitosan remains underexplored. This study aims to contribute to the broader understanding of processing–structure relationships and thereby support the optimized utilization of insect chitosan in diverse applications.

## 2 Materials and methods

### 2.1 Materials

TM, PB, and AD larvae were purchased from a local insect farm (Jeongeup, Republic of Korea). Larvae from each species (∼500 g) were collected, stored at −20 °C, and subsequently prepared for drying and chitosan extraction. Shrimp shell–derived chitosan (DD = 75 %, Sigma-Aldrich) was used as a control. 2,2-Diphenyl-1-picrylhydrazyl (DPPH) and 2,2′-azino-bis(3-ethylbenzothiazoline-6-sulfonic acid) (ABTS) were purchased from Sigma-Aldrich (MO, USA). Ethanol, acetic acid, HCl, NaOH pellets, H_2_O_2_, and sodium acetate were purchased from Daejung (Siheung, Republic of Korea).

### 2.2 Extraction of chitosan

Chitosan extraction was carried out using a modification of the method of Luo et al. (2019). The lar*v*ae were immersed into ethanol for 1 h, thoroughly rinsed with distilled water, and then subjected to hot-air drying at 60 °C for 48 h or to freeze-drying at −50 °C for 96 h using a con*v*entional freeze dryer (Ilshin, Republic of Korea). The dried materials were ground to pass through a 100-mesh sieve. The ground samples were stirred in *n*-hexane (1:5 *w*/*v*) at room temperature for 1 h, and the lipid-containing sol*v*ent was decanted. This step was repeated five times until the supernatant became clear. The remaining sol*v*ent was evaporated under a fume hood o*v*er 12 h. The defatted material was transferred into 1 M aqueous HCl (1:15 *w*/*v*) and kept at 30 °C under continuous agitation (200 rpm) for 2 h to accomplish decalcification. The mixture was filtered, and the residue was washed to neutral pH and dried at 60 °C. Deproteinization was performed using a modification of the method of Jiang et al. (2023) by stirring in 4 M NaOH (1:15, w/v) at 90 °C for 6 h. Subsequently, decolorization was carried out using 10 % H_2_O_2_ (1:40, w/v) at 80 °C for 30 min. Finally, deacetylation was performed using 40 % NaOH (1:15, w/v) at 95 °C for 8 h following the method of da Silva et al. (2021). After deacetylation, chitosan was filtered, neutralized, and dried. Identical extraction and deacetylation parameters were applied across all species to ensure methodological consistency, as this study primarily aimed to examine the impact of drying pretreatment rather than establish optimized protocols for individual insects. The chitosan yield was calculated as

(1) $$Y\text{ield}\;\left(\%\right)=\frac{\mathrm{Dried}\;\text{chitosan}\;\mathrm{mass}\;\left(g\right)}{R\mathrm{aw}\;\text{material}\;\mathrm{mass}\;\left(g\right)}\times 100$$

The samples were denoted as X_Y, where X denotes the drying method (HD = hot-air-dried, FD = freeze-dried) and Y denotes the sample origin (TM, PB, or AD).

### 2.3 Characterization

#### 2.3.1 Solubility

Chitosan (0.1 g) was dispersed in 1 % aqueous acetic acid (10 mL) inside a centrifuge tube. The mixture was stirred at 30 °C for 1 h and then centrifuged (3500 *g*, 10 min). The supernatant was removed, and the pellet was dried overnight. Solubility was calculated as

(2) $$\mathrm{Solubility}\;\left(\%\right)=\frac{M_{1}-M_{2}}{M_{1}-M_{0}}\times 100,$$

where *M*_0_ is the initial weight of the tube, *M*_1_ is the combined weight of the tube and chitosan, and *M*_2_ is the final weight of the tube after drying.

#### 2.3.2 Water binding capacity (WBC) and fat binding capacity (FBC)

The WBC and FBC were measured as described by Luo et al. (2019). For WBC determination, chitosan (0.5 g) was placed in a preweighed centrifuge tube and suspended in distilled water (10 mL) by vortexing for 1 min. The mixture was left to stand for 30 min with shaking for 5 s every 10 min. After centrifugation at 1500 *g* for 25 min, the supernatant was discarded, and the tube was weighed. FBC determination followed the same procedure except for the use of soybean oil instead of distilled water. The WBC and FBC were calculated as

(3) $$\mathrm{WBC}\;\left(\%\right)=\frac{B\text{ound}\;\mathrm{water}\;\text{weight}\;\left(g\right)}{I\text{nitial chitosan weight}\;\left(g\right)},$$

(4) $$\mathrm{FBC}\;\left(\%\right)=\frac{B\text{ound}\;\mathrm{fat}\;\text{weight}\;\left(g\right)}{I\text{nitial chitosan weight}\;\left(g\right)}.$$

#### 2.3.3 Moisture and ash contents

Chitosan (0.5 g) was oven-dried to a constant weight at 105 °C, cooled, and weighed. The moisture content was calculated as

(5) $$\text{Moisture}\;\left(\%\right)=\frac{I\text{nitial weight}\;\left(g\right)-\text{dried weight}\;\left(g\right)}{I\text{nitial weight}\;\left(g\right)}\times 100.$$

A crucible was charged with chitosan (0.5 g), heated at 600 °C in a muffle furnace for 6 h, cooled, and weighed. The ash content was calculated as

(6) $$\mathrm{Ash}\;\left(\%\right)=\frac{R\text{esidual weight}\;\left(g\right)}{I\text{nitial weight}\;\left(g\right)}\times 100.$$

#### 2.3.4 Molecular weight

The molecular weight of chitosan was estimated using intrinsic viscosity (*η*) measurements. Chitosan was dissolved in a 0.5 M acetic acid/0.5 M sodium acetate buffer to concentrations of 0.0010, 0.0015, 0.0020, and 0.0050 g/mL. The specific viscosity (*η*_sp_) was determined at 25 °C using a capillary viscometer by comparison with a blank solvent. The intrinsic viscosity was obtained from the intercept of the reduced viscosity (*η*_red_) versus concentration plot. The viscosity-average molecular weight (*M*_v_) was then estimated using the Mark–Houwink equation:

(7) $$\eta =K{M_{v}}^{a},$$

where *K* and *a* are constants depending on the solute, solvent properties, and experimental temperature. *K* = 0.119 and *a* = 0.59 were used in our calculations.

#### 2.3.5 DD

The DD was determined using potentiometric titration according to the method of da Silva et al. (2021). Chitosan (0.1 g) was added to 0.1 M aqueous HCl and stirred for 30 min. Subsequently, distilled water (25 mL) was added, and the mixture was stirred until the chitosan was completely solubilized. The resulting solution was titrated with standardized 0.1 M NaOH. A titration curve was obtained by plotting the volume of consumed NaOH (Δ*V*) against pH, and the DD was calculated as

(8) $$\mathrm{DD}\;\left(\%\right)=2.03\times \frac{∆V}{w+\left(0.0042\times ∆V\right)},$$

where *w* is the sample weight.

#### 2.3.6 X-ray diffraction (XRD) analysis

XRD measurements (D8 Advance, Bruker) were performed using Cu *K*_α_ radiation at 40 mA and 40 kV. Data were collected over a scan range of 5°–80° at a scan rate of 10°/min. The crystallinity index (CrI) was calculated as

(9) $$\mathrm{CrI}\;\left(\%\right)=\left(\left(\frac{I-I_{\mathrm{am}}}{I}\right)\times 100\right),$$

where *I* is the maximum intensity at 20°, and *I*_am_ is the maximum intensity of the amorphous-phase peak at 16°.

#### 2.3.7 Fourier transform infrared (FT-IR) spectroscopy

FT-IR spectra (Thermo Scientific Nicolet iS10 FT-IR spectrometer) were recorded using the KBr pellet method o*v*er a wavenumber range of 500–4000 cm^−1^ at a resolution of 4 cm^−1^. The final spectrum was obtained by averaging 32 scans.

#### 2.3.8 Thermogravimetric analysis (TGA)

For TGA (TGA Q5000 IR/SDT Q600), chitosan (3 mg) was heated from 25 °C to 600 °C at a rate of 5 °C/min under a nitrogen flow of 100 mL/min.

#### 2.3.9 Rheological properties

Chitosan was dissolved in 1 vol% aqueous acetic acid to a concentration of 2 % (*w*/*v*), and the solution was stirred for 3 h. Undissolved chitosan particles and air bubbles were removed by centrifugation at 9000 *g* for 5 min, and the rheological properties of the resulting solution were determined using a modular compact rheometer (MCR 102, Anton Paar, Australia) equipped with a parallel plate (PP40, gap = 0.1 mm).

The flow curve was evaluated at 25 °C in a shear rate range of 1–1000 s^−1^. The linear viscoelastic region (LVER) was determined using an amplitude sweep test at 1 Hz and strains of 1 %–100 %. The frequency sweep–based storage modulus (*G*′) and loss modulus (*G*″) determinations were performed at 25 °C and a fixed strain of 2 % over a frequency range of 0.1–10 Hz. Temperature-dependent viscoelastic properties were assessed by heating from 25 °C to 80 °C at 1 Hz and 2 % strain.

#### 2.3.10 Field-emission scanning electron microscopy (FE-SEM)

The surface morphology of chitosan was observed using FE-SEM (S-4700, Hitachi). Images were captured at an accelerating voltage of 10 kV for gold-coated samples.

#### 2.3.11 DPPH assay

The DPPH radical scavenging assay was performed using chitosan solutions with concentrations of 0.5–10 mg/mL. The sample (20 μL) and 0.1 mM ethanolic DPPH (180 μL) solutions were mixed in a 96-well plate. Ethanol was used as the blank under the same conditions. After incubation in the dark for 2 h, the absorbance of each well was measured at 515 nm using a spectrophotometer (SpectraMax M2e, Molecular Devices, USA). The DPPH radical scavenging activity was calculated as

(10) $$\text{DPPH radical scaveng}\mathrm{ing}\;\text{activity}\;\left(\%\right)=\left(1-{\mathrm{OD}}_{\text{sample}}/{\mathrm{OD}}_{\text{blank}}\right)\times 100,$$

where OD_sample_ is the absorbance of the sample and OD_blank_ is the absorbance of the blank.

#### 2.3.12 ABTS assay

A 14.8 mM ABTS solution in a 0.1 M phosphate buffer was mixed with an equal volume (1 mL) of 5 mM potassium persulfate. The mixture was incubated in the dark at room temperature for 16 h. The resulting solution was diluted with distilled water to adjust the absorbance at 734 nm to 0.70 ± 0.05. The ABTS (180 μL) and sample (20 μL) solutions were mixed in a 96-well plate. Distilled water was used as the blank. After incubation for 1 h at room temperature, absorbance at 734 nm was measured using a spectrophotometer. The ABTS radical scavenging activity was calculated as

(11) $$\text{ABTS radical scaveng}\mathrm{ing}\;\text{activity}\;\left(\%\right)=\left(1-{\mathrm{OD}}_{\text{sample}}/{\mathrm{OD}}_{\text{blank}}\right)\times 100,$$

where OD_sample_ is the absorbance of the sample and OD_blank_ is the absorbance of the blank.

### 2.4 Statistical analysis

Statistical analysis was performed using the IBM SPSS Statistics 20 software (IBM Corp., Chicago, IL, USA), and significant differences were determined using the one-way analysis of variance and Tukey's post hoc test. Multivariate statistical analysis (multiple factor analysis) was performed using the FactoMineR package (Lê et al., 2008) implemented in RStudio 4.4.2 (R Core Team, Vienna, Austria), with the significance level set at *p* < 0.05. All analyses were performed in triplicate (*n* = 3) using repeated measurements of the same extracted sample, and the results were expressed as the corresponding means ± standard deviations.

## 3 Results and discussion

### 3.1 Yield

The extraction yield depended on the insect species and was highest for AD (*p* < 0.05) (Table 1). The lower yield observed for TM was attributed to the removal of ∼30 % of lipids in the defatting stage. After demineralization, 41.52 %–55.98 % of the original weight was retained, while 13.31 %–25.64 % of the original weight was retained after deproteinization. Decolorization led to a mass loss of 10 %, and deacetylation resulted in mass losses of 51.14 %–72.58 %. These variations in yield were attributed to the differing protein and lipid contents of the insect species, and several studies have also reported factors influencing the yields of chitosan extraction from insects (Luo et al., 2019; Mohan et al., 2020). Additionally, the particularly high yield of AD may be associated with its thicker or more developed cuticle, which could provide a larger chitin reservoir for conversion (Muthukrishnan et al., 2020). Previous studies have also reported that yields can vary with the developmental stage of TM (Chalghaf et al., 2023), suggesting that biological characteristics contribute to interspecies variability. Furthermore, differences between hot-air-dried and freeze-dried samples within the same species indicate that drying pretreatment can modulate recovery efficiency, although its effect appears to be species-dependent.

**Table 1:** Physicochemical properties of different chitosan samples denoted as X_Y, where X denotes the drying method (HD = hot-air-dried, FD = freeze-dried) and Y denotes the sample origin (TM = *Tenebrio molitor*, PB = *Protaetia brevitarsis*, or AD = *Allomyrina dichotoma*).

Sample	Yield (%)	Solubility (%)	WBC (%)	FBC (%)	Moisture (%)	Ash (%)
HD_PB	4.42 ± 0.49^c^	52.06 ± 1.35^d^	456.06 ± 12.40^d^	316.57 ± 4.94^e^	0.69 ± 0.01^e^	0.16 ± 0.02^de^
HD_AD	7.59 ± 0.06^a^	70.20 ± 1.41^c^	379.63 ± 9.68^d^	203.83 ± 0.06^f^	1.15 ± 0.03^c^	0.45 ± 0.02^b^
HD_TM	2.20 ± 0.13^e^	94.05 ± 0.67^a^	842.71 ± 20.33^b^	725.23 ± 21.68^b^	0.80 ± 0.07^de^	0.22 ± 0.01^d^
FD_PB	3.14 ± 0.20^d^	76.06 ± 0.70^b^	935.56 ± 36.09^b^	643.81 ± 20.95^c^	1.27 ± 0.02^bc^	0.45 ± 0.01^b^
FD_AD	5.72 ± 0.31^b^	68.64 ± 2.55^c^	640.50 ± 33.70^c^	354.86 ± 14.27^d^	1.61 ± 0.05^a^	1.17 ± 0.05^a^
FD_TM	2.17 ± 0.17^e^	94.12 ± 0.76^a^	1088.54 ± 70.02^a^	833.66 ± 5.11^a^	1.37 ± 0.03^b^	0.37 ± 0.01^c^
Shrimp	–	94.45 ± 1.28^a^	917.46 ± 17.40^b^	238.73 ± 7.70^f^	0.87 ± 0.05^d^	0.14 ± 0.01^e^

All values are represented as means ± standard deviations (*n* = 3). ^a–f^Different superscript letters within a column indicate significant differences (*p* < 0.05).

### 3.2 Solubility

The solubility of chitosan ranged from 52.06 % to 95.39 % (Table 1) and was highest for shrimp-derived chitosan followed by TM-derived chitosan (*p* < 0.05). Drying pretreatment exerted a pronounced influence in the case of PB, with HD_PB and FD_PB exhibiting solubilities of 52.06 % and 76.06 %, respectively. Solubility variations have generally been attributed to the DD, as higher DDs increase the availability of amino groups and enhance hydrophilicity (Kapadnis et al., 2019). *M*_v_ also affects solubility, as it is positively correlated with the proportion of insoluble high-molecular-weight fractions (Sogias et al., 2010). In this study, however, the CrIs of HD_PB and FD_PB were nearly identical (30.16 % vs. 30.52 %, Table 2), indicating that crystallinity was not a decisive factor. FE-SEM analysis showed that FD_PB exhibited a rougher texture with irregular cracks compared with the multilayered wrinkled structure of HD_PB (Fig. 5), which may have facilitated solvent penetration during dissolution. Taken together, these results suggest that solubility was primarily influenced by the DD and *M*_v_, while surface morphology associated with drying pretreatment may have also contributed to the observed differences.

**Table 2:** Molecular weights, deacetylation degrees, and crystallinity indices of different chitosan samples.

Samples	*M*_v_ (Da)	DD (%)	CrI (%)
HD_PB	(7.874 ± 0.383) × 10^3 d^	43.14 ± 1.20^d^	30.16 ± 2.41^bc^
HD_AD	(24.874 ± 1.645) × 10^3 d^	70.86 ± 2.04^c^	39.09 ± 2.59^a^
HD_TM	(22.422 ± 0.840) × 10^4 b^	85.11 ± 1.19^a^	18.72 ± 0.72^d^
FD_PB	(24.407 ± 0.821) × 10^4 b^	85.60 ± 0.74^a^	30.52 ± 0.84^bc^
FD_AD	(14.347 ± 0.396) × 10^4 c^	72.47 ± 2.08^bc^	27.26 ± 0.75^c^
FD_TM	(23.274 ± 1.160) × 10^4 b^	89.12 ± 0.21^a.^	16.39 ± 0.45^d^
Shrimp	(16.637 ± 0.901) × 10^5 a^	75.96 ± 0.77^b^	33.68 ± 0.47^b^

All values are represented as means ± standard deviations (*n* = 3). ^a–d^Different superscript letters within a column indicate significant differences (*p* < 0.05).

### 3.3 WBC and FBC

The WBC was highest for FD_TM (1088 %). WBCs comparable with that of shrimp-derived chitosan (917 %) were observed for FD_PB (935 %) and HD_TM (842 %) (Table 1). Overall, the WBC decreased in the order of TM > PB > AD. Furthermore, freeze-drying significantly increased the WBC of insect-derived chitosan (*p* < 0.05). The WBC is associated with pore size and molecular structure, and hydrophilic interactions can be regulated through intra- and intermolecular hydrogen bonding (Ahmadi et al., 2019; Mirhosseini & Amid, 2012). Freeze-drying facilitates the formation of a porous structure and, hence, the production of a hydrophilic porous polymer, with the increased porosity further enhancing hydration properties (Guadalupe-Moyano et al., 2022; Qian & Zhang, 2011).

The FBC was highest for FD_TM (833 %) and lowest for shrimp-derived chitosan. In the case of insect-derived chitosan, the FBC decreased in the order of TM > PB > AD, following the pattern observed for the WBC. Freeze-drying significantly increased the FBC (*p* < 0.05). These findings agree with those of previous studies, demonstrating that freeze-drying increased both the WBC and FBC (Li et al., 2019; Miah et al., 2025; Shen et al., 2021) and suggesting that binding property differences were contingent on the drying method and insect species. The observed differences suggest that physicochemical modifications induced by drying may play a role in shaping the structural and functional properties of chitosan.

### 3.4 Moisture and ash contents

The moisture content was highest for FD_AD (1.61 %), with the second and third highest values observed for FD_TM (1.37 %) and FD_PB (1.27 %), respectively (Table 1). The moisture content of shrimp-derived chitosan was similar to that of HD_TM. Similar to the WBC and FBC, freeze-dried samples exhibited notably higher values (*p* < 0.05). As reported by Gu et al. (2022) and Pissia et al. (2022), freeze-dried powders are likely to have high rehydration rates because of their highly porous structures. Considering that chitosan extraction involved the use of acids and bases as a part of the rehydration process, we inferred that high rehydration rates may contribute to the increased moisture content.

The ash content was highest for FD_AD (1.17 %) and was less than 1 % for all other samples (Table 1). This parameter is a key indicator for evaluating the purity of chitosan (Abdel-Rahman et al., 2015), with values below 1 % indicating high quality (Ibitoye et al., 2018). The ash content of insect-derived chitosan depended on the insect species, which was ascribed to the species-dependent nature of the mineral composition of the exoskeleton.

### 3.5 *M*_v_

The *M*_v_s of different samples are listed in Table 2. The *M*_v_ of shrimp-derived chitosan (16.637 × 10^5^ Da) significantly exceeded that of insect-derived chitosan (*p* < 0.05), which was species-dependent. For TM chitosan, no significant difference was observed between drying methods, whereas for PB chitosan, FD_PB exhibited a significantly higher *M*_v_ than HD_PB. This finding was attributed to the lower solubility of HD_PB, which probably resulted in a lower *M*_v_. This outcome may be attributed to the fact that freeze-drying avoids prolonged thermal exposure and thus reduces the likelihood of depolymerization and helps maintain chain integrity, unlike hot-air drying (Zhang et al., 2024). In addition, studies on crustacean-derived chitosan have shown that the choice of drying route can alter molecular weight and crystalline organization, indicating that drying processes influence the physicochemical properties of chitosan (Arantes et al., 2015). Generally, the molecular weight of commercial chitosan ranges from 50 to 2000 kDa and is classified as low (≤100 kDa), medium (100–1000 kDa), and high (≥1000 kDa) (Gonçalves et al., 2021). Herein, the *M*_v_s of HD_PB and HD_AD were categorized as low, while those of the remaining insect-derived samples were classified as medium. Low-molecular-weight chitosan exhibits advantageous biological properties, including antioxidant and antimicrobial activities and biodegradability, and forms low-viscosity aqueous solutions, which may enhance permeability (Román-Doval et al., 2023).

### 3.6 DD

The DD is an important parameter that influences the physicochemical and biological properties of chitosan (e.g., viscosity, solubility, and molecular weight) and acts as an indicator for determining its classification and potential applications (da Silva et al., 2021; Keerthika & Jayakumar, 2024). The DD of insect-derived chitosan depended on the species and drying method, with the highest (89.12 %) and lowest (43.14 %) values observed for FD_TM and HD_PB, respectively (Table 2). In general, most chitosan samples exhibited DDs exceeding 70 %. The DD of shrimp-derived chitosan (75.96 %) was close to that specified by the manufacturer (≥75 %). The drying method had no significant effect on the DD of TM and AD chitosan. However, FD_PB demonstrated a significantly increased DD (*p* < 0.05). This difference may be associated with structural modifications induced by freeze-drying, which could increase the accessibility of reactive sites to alkali and thereby enhance deacetylation. When viewed together with the *M*_v_ data (Section 3.5), these results point to the possibility that drying pretreatment influences the structural reactivity of chitin, which has potential implications for the *M*_v_ and DD. The DD is generally influenced by various processing parameters, including the extraction method, reaction temperature and time, and NaOH concentration (Triunfo et al., 2022).

### 3.7 XRD analysis

**Fig. 1:**
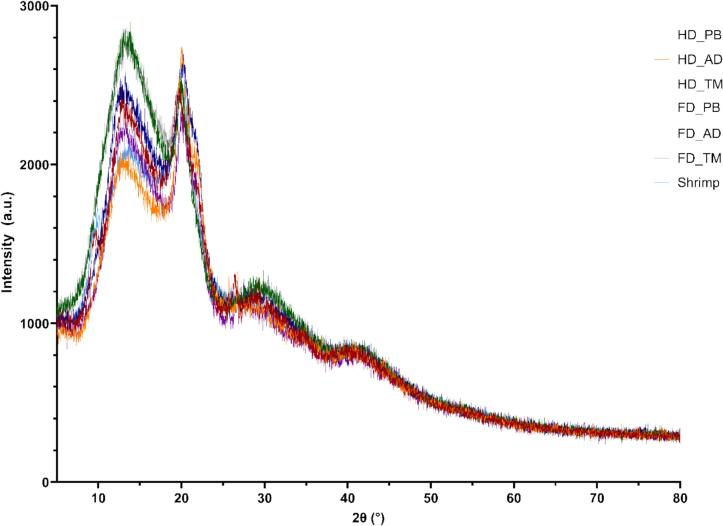
X-ray diffraction patterns of different chitosan samples.

The XRD patterns of chitosan (Fig. 1) featured the characteristic peaks of Form I and Form II structures at approximately 12° and 20°, respectively (Ma et al., 2015; Trung et al., 2006), with the CrI values (16.39 %–39.09 %) indicating low crystallinities. The DD is generally considered to be negatively correlated with crystallinity. A similar pattern was observed for TM chitosan, which featured a high DD and low crystallinity. Low-crystallinity chitosan is well suited for heavy metal adsorption, possibly because of the porous structure formed during freeze-drying (Qi & Xu, 2004).

### 3.8 FT-IR spectroscopy

**Fig. 2:**
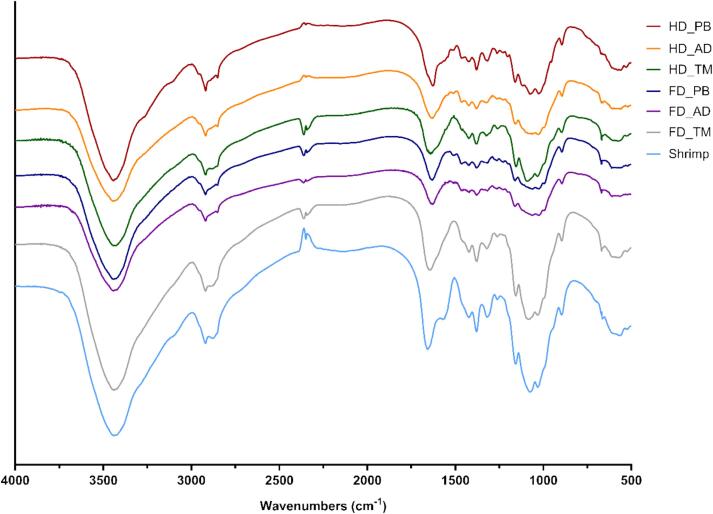
Fourier transform infrared spectra of different chitosan samples.

Fig. 2 presents the FT-IR spectra of insect-derived chitosan. The broad absorption band at 3440 cm^−1^ was attributed to the stretching vibrations of N—H and O—H bonds, and the peaks at 2918 and 2360 cm^−1^ corresponded to the asymmetric stretching of CH_2_ groups in CH_2_OH moieties. The region between 1500 and 1700 cm^−1^, which is widely used to distinguish between chitin and chitosan (da Silva et al., 2021), included a characteristic C=O stretch at 1636–1654 cm^−1^ (amide I band). The peak at 1378 cm^−1^ was assigned to C—N vibrations (amide III band). The weak peak at 1420 cm^−1^ corresponded to CH_2_ bending. The peak at 1315 cm^−1^ was assigned to C—N stretching, and the peaks in the 1061–1073 cm^−1^ region were ascribed to C–O–C stretching vibrations. The peaks at 894 and 1156–1160 cm^−1^ were attributed to the β-(1 → 4)-glycosidic bonds of the chitosan backbone (Li et al., 2019; Ma et al., 2015). The FT-IR spectra of shrimp- and insect-derived chitosan exhibited similar peak positions while featuring minor differences in peak intensities.

### 3.9 TGA

**Fig. 3:**
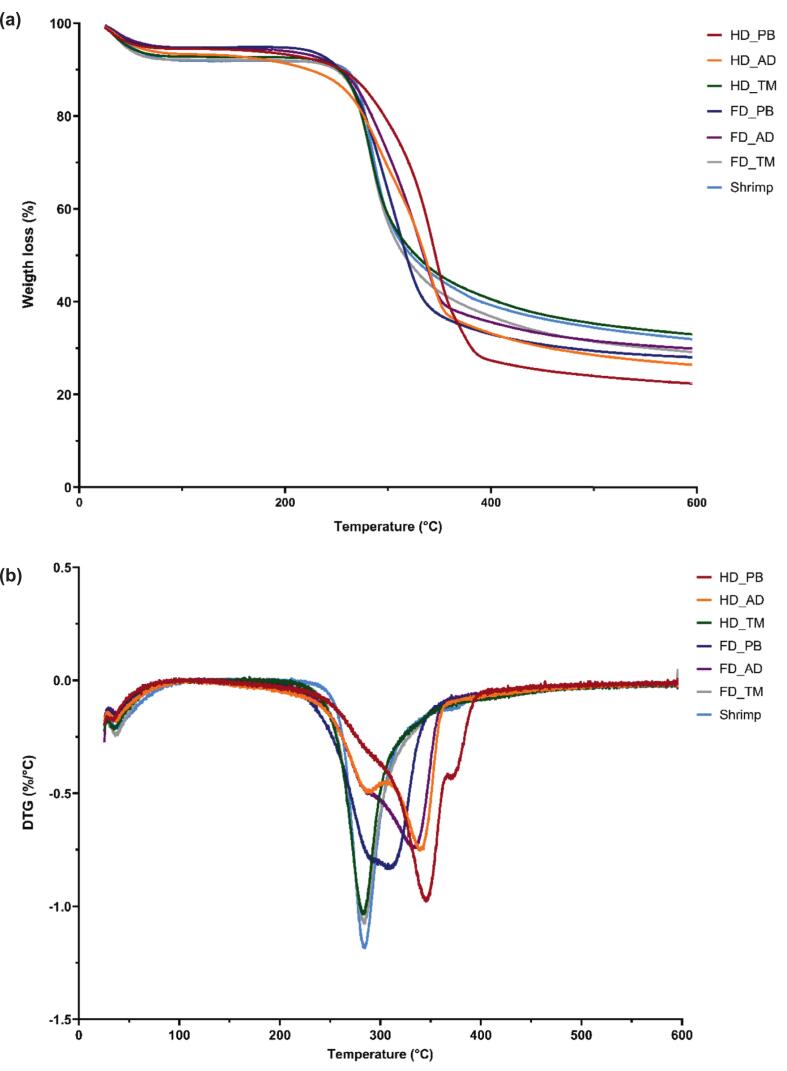
(a) Thermogravimetric analysis and (b) differential thermogravimetry curves of different chitosan samples.

The thermal degradation of insect-derived chitosan (Fig. 3a) featured two stages (Neto et al., 2005). The first stage (25–125 °C) was characterized by moisture evaporation and a mass loss of 4.01 %–8.27 %, with the highest mass loss observed for shrimp-derived and TM chitosan. The second stage was characterized by a major mass loss of 59.27 %–73.90 % up to ∼600 °C. This mass loss was highest for PB chitosan and attributed to the thermal decomposition of the chitosan backbone, which involved the dehydration of saccharide rings and depolymerization of deacetylated units (Paulino et al., 2006). The residual mass after thermal decomposition ranged from 21.53 % to 33.63 %.

Differential thermogravimetry (DTG) peaks represent the maxima of the mass loss rate during thermal degradation. Herein, these peaks were observed around 284 °C for shrimp-derived and TM chitosan, while HD_PB and HD_AD showed peaks at ∼342 °C (Fig. 3b). In contrast, FD_PB and FD_AD featured peaks at ∼310 and 332 °C, respectively. Thus, the thermal behavior was influenced by the drying method, even for samples derived from the same insect species. PB and AD chitosan exhibited the highest thermal stabilities, particularly when produced from hot-air-dried specimens. This enhanced stability was attributed to differences in the DD, *M*_v_, and crystallinity, which strongly influence the thermal behavior of chitosan (Zhu et al., 2023).

### 3.10 Rheological properties

**Fig. 4:**
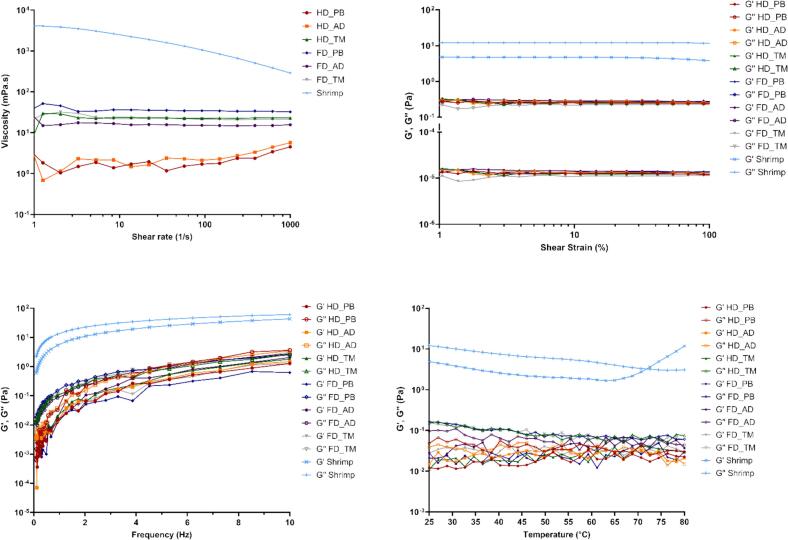
Rheological properties of different chitosan samples: (a) flow curves, (b) strain sweep curves, (c) angular frequency sweep curves, (d) temperature ramp curves. *G*′: storage modulus; *G*″: loss modulus.

The rheological properties of different chitosan samples are presented in Fig. 4. Fig. 4a presents viscosity responses across varying shear rates (1–1000 s^−1^). Shrimp-derived chitosan exhibited shear-thinning behavior (i.e., its viscosity decreased with the increasing shear rate), behaving as a non-Newtonian fluid (Enoch & Somasundaram, 2024). In contrast, insect-derived chitosan displayed lower viscosities that were nearly constant across the shear rate range, exhibiting a Newtonian-like behavior. The apparent viscosity followed the order of FD_PB, FD_TM, HD_TM, and FD_AD, which aligned with the *M*_v_ order of the respective samples. Notably, HD_AD and HD_PB had the lowest molecular weights and viscosities.

The results of LVER determination (Fig. 4b) indicated that in all cases, *G*″ exceeded *G*′, suggesting liquid-like behavior (dos Santos Carvalho et al., 2022). *G*′ and *G*″ remained relatively constant with the increasing strain, which confirmed that the measurements were performed within the LVER. Insect-derived chitosan exhibited lower *G*′ and *G*″ values than shrimp-derived chitosan, thus featuring lower viscosities and structural stabilities (Luo et al., 2019).

The dynamic viscoelastic properties of chitosan were evaluated through a frequency sweep test (Fig. 4c). *G*′ and *G*″ increased with the increasing frequency, and *G*″ remained higher than *G*′ in all cases. This finding indicated that viscous behavior dominated over elastic behavior and agreed with liquid-like rheological characteristics (Hamdi et al., 2019). Shrimp-derived chitosan exhibited *G*′ and *G*″ values significantly higher than those of insect-derived chitosan, which was ascribed to the increased chain entanglement due to the higher molecular weight of the former. Molecular weight is generally recognized as a determinant of chitosan viscosity. Chitosan with lower molecular weights exhibits reduced chain entanglement, thus showing greater molecular flexibility and lower viscosity (Arancibia et al., 2015).

The temperature-dependent rheological behaviors of chitosan solutions at 25–80 °C are shown in Fig. 4d. For shrimp-derived chitosan, *G*″ decreased with the increasing temperature, whereas *G*′ increased starting from 65 °C. The gelation temperature was identified as 71.9 °C and corresponded to the point where *G*′ equaled *G*″, indicating the onset of gel formation and the transition to hydrogel-like behavior at higher temperatures (dos Santos Carvalho et al., 2022). In contrast, insect-derived chitosan generally showed *G*″ > *G*′ and thermally unstable curves. This behavior was attributed to the lower viscosity and more distinct liquid-like characteristics of these samples.

### 3.11 FE-SEM analysis

**Fig. 5:**
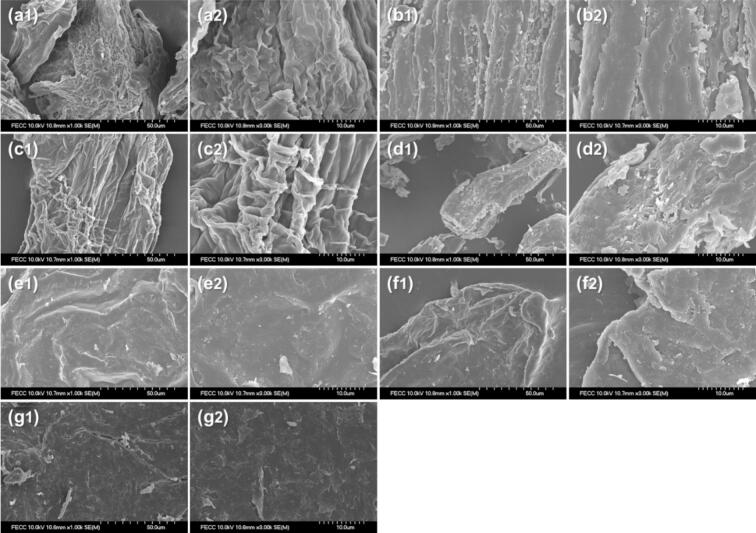
Scanning electron microscopy images of (a) HD_PB, (b) HD_AD, (c) HD_TM, (d) FD_PB, (e) FD_AD, (f) FD_TM, and (g) shrimp-derived chitosan. Indices 1 and 2 denote 1000× and 3000× magnifications, respectively.

HD_PB exhibited a multilayered wrinkled structure with entangled surfaces (Fig. 5a), while FD_PB (Fig. 5b) showed a rough texture with irregular cracks. HD_AD (Fig. 5c) displayed an irregular overlapping structure, whereas FD_AD (Fig. 5d) featured a coarse surface with visible cracks. HD_TM (Fig. 5e) had a relatively smooth and flat surface, whereas FD_TM (Fig. 5f) exhibited a rougher morphology. Shrimp-derived chitosan (Fig. 5g) showed a heterogeneous and rough surface. Overall, diverse surface morphologies were observed, depending on the insect species and drying method, with freeze-dried samples generally exhibiting higher surface roughness.

### 3.12 Antioxidant activity of insect-derived chitosan

**Fig. 6:**
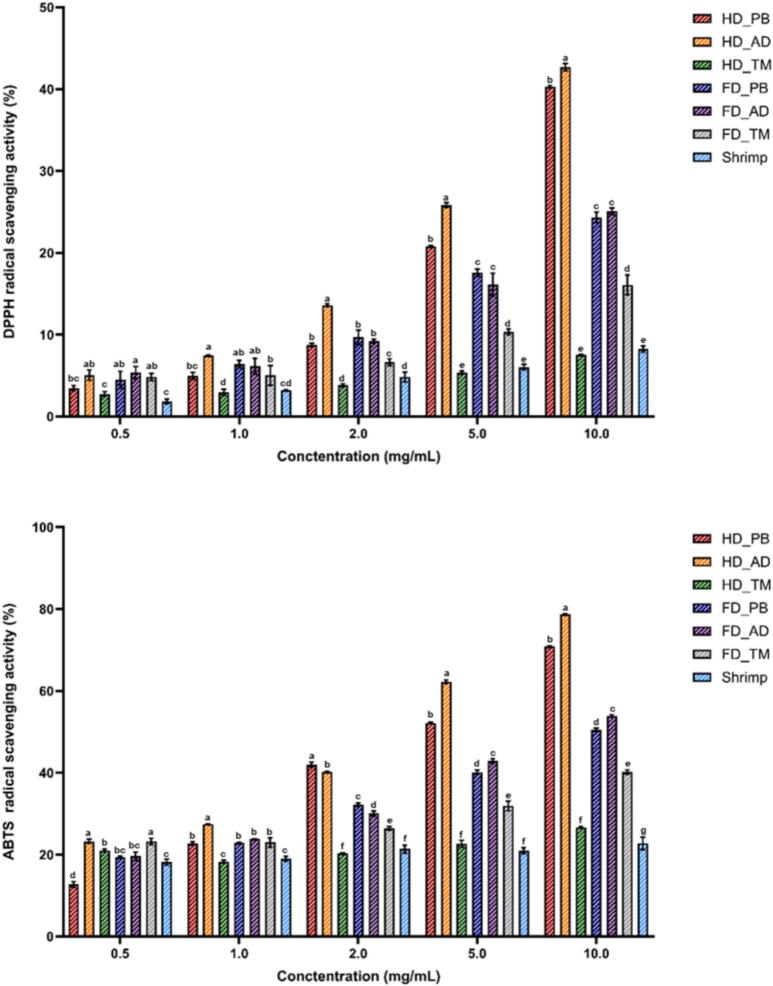
(a) Diphenyl-1-picrylhydrazyl and (b) 2,2′-azino-bis(3-ethylbenzothiazoline-6-sulfonic acid) radical scavenging activities of different chitosan samples. Different superscript letters within a column indicate significant differences (*p* < 0.05).

The DPPH and ABTS radical scavenging activities (Fig. 6) increased with the increasing concentration. The antioxidant activity of chitosan is closely associated with its hydrogen-donating capacity (Tamer et al., 2023). HD_AD exhibited the highest DPPH radical scavenging activity, which reached 42.69 % at a concentration of 10 mg/mL, whereas HD_TM showed the lowest activity (7.53 %). Similarly, the ABTS scavenging activity was highest for HD_AD (78.54 %) and lowest for shrimp-derived chitosan (22.76 %). These results indicate that the antioxidant activity of chitosan was substantially influenced by the insect species. The high activity of HD_AD coincided with its low *M*_v_ (24.874 × 10^3^ Da), suggesting that molecular size may have influenced this behavior. This observation aligns with previous reports indicating that low-molecular-weight chitosan exhibits enhanced radical scavenging activity (Kim & Thomas, 2007) and is further supported by recent findings showing similar trends (Psarianos et al., 2025). The ABTS scavenging activity exceeded the DPPH scavenging activity in all cases, which was attributed to the fact that ABTS generates cationic radicals, while DPPH is a neutral free radical (Lee et al., 2022). In addition, as ABTS and DPPH were used in aqueous and ethanolic solutions, respectively, differences in the dissolution environment can also be responsible for the antioxidant activity variation (Liu et al., 2024).

### 3.13 Correlations among different properties

**Fig. 7:**
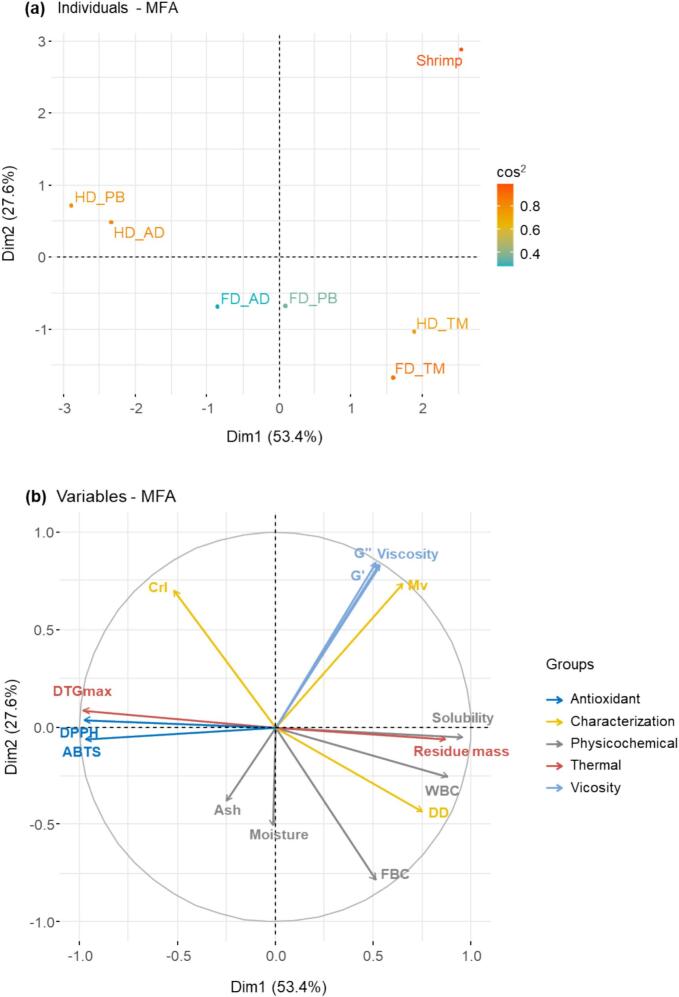
Multiple factor analysis plots of different chitosan samples: (a) individuals, (b) variables.

Relationships between different chitosan properties were established using multiple factor analysis (Fig. 7). Dimension 1 (Dim 1) and Dimension 2 (Dim 2) accounted for 53.4 % and 27.6 % of the total variance, respectively, with their combined contribution equaling 81.0 %. Shrimp and TM chitosan, positioned in the positive direction of Dim 1, showed strong positive correlations with the DD, *M*_v_, and viscosity. In contrast, HD_PB and HD_AD, located in the negative direction of Dim 1, exhibited positive correlations with antioxidant activity (DPPH and ABTS) and thermal stability (DTG peak position). FD_AD and FD_PB were positioned near the center of the plot, which was indicative of intermediate characteristics without a strong correlation with specific variables. Dim 1 predominantly represented structural characteristics, with high contributions observed for the DD, *M*_v_, and viscosity, whereas Dim 2 primarily accounted for the variance associated with thermal and antioxidant parameters, including the DTG peak position and radical scavenging activity (Yaich et al., 2017). In the variable plot, *G*′ and *G*″ were positioned along similar directions, which indicated a strong positive correlation between the viscoelastic parameters and aligned with the trend observed for *M*_v_, suggesting that higher *M*_v_ contributes to enhanced viscoelastic behavior (Luo et al., 2019; Qian et al., 2023). In contrast, the DD and CrI were positioned in opposite directions, which suggested a negative correlation between these parameters (Qi & Xu, 2004). Shrimp-derived chitosan was clearly separated from insect-derived samples, which may reflect its commercial processing and relatively high *M*_v_, viscosity, and structural stability. For TM chitosan, the freeze-dried and hot-air-dried groups clustered closely together, which indicated minimal structural changes due to drying. In contrast, PB and AD chitosan formed distinct clusters based on the drying method, which suggested that hot-air drying could have reduced the DD and *M*_v_ while enhancing the antioxidant activity.

## 4 Conclusion

Chitosan was extracted from the larvae of three edible insects (PB, AD, and TM) subjected to different drying pretreatments and compared with commercial shrimp-derived chitosan in terms of physicochemical and structural properties. The species and drying method significantly influenced the yield, DD, *M*_v_, solubility, antioxidant activity, and structural features. Hot-air-dried PB and AD produced low-*M*_v_ chitosan with enhanced antioxidant activity, whereas TM yielded a material with characteristics similar to those of shrimp-derived chitosan. These findings highlight the potential of edible insects as sustainable alternative sources of chitosan and underscore the importance of pretreatment conditions for determining functional properties. Several practical challenges must be addressed for industrial feasibility, including differences in breeding costs among insect species that influence economic competitiveness, the need to optimize extraction processes to reduce chemical use and improve efficiency at scale, and the requirement to ensure the batch-to-batch stability of purity and functionality to meet regulatory and commercial standards. Addressing these aspects will be critical for translating laboratory-scale findings into viable applications.

## CRediT authorship contribution statement

**Ji Yoon Cha:** Writing – review & editing, Writing – original draft, Formal analysis, Data curation, Conceptualization. **Yea-Ji Kim:** Writing – review & editing, Writing – original draft, Formal analysis. **Jeong Heon Kim:** Writing – review & editing, Writing – original draft, Methodology, Formal analysis. **Dong-Hyun Keum:** Writing – review & editing, Writing – original draft, Methodology, Formal analysis. **Jaejoon Han:** Writing – review & editing, Writing – original draft, Validation, Methodology. **Yun-Sang Choi:** Writing – review & editing, Writing – original draft, Validation, Supervision, Methodology, Investigation, Formal analysis, Conceptualization.

## Declaration of competing interest

The authors declare that they have no known competing financial interests or personal relationships that could have appeared to influence the work reported in this paper.

## Data Availability

No data was used for the research described in the article.
